# Pulmonary Involvement in Crohn’s Disease: A Rare Case Report

**DOI:** 10.7759/cureus.2710

**Published:** 2018-05-30

**Authors:** Yasar Sattar, Zarafshan Zubair, Nirav B Patel, Faiqa S Zafar, Ali Hassan, Nargis Tariq, Sharaad Latchana, Sharmi Biswas, Norina Usman, Stefany C Lopez Pantoja

**Affiliations:** 1 Research Assistant, Kings County Hospital Center, New York, USA; 2 MBBS, Dow University of Health Sciences (DUHS), Karachi, Pakistan; 3 Department of Medicine, Lasante Health; 4 Medical Graduate, American University of Antigua; 5 Medical Graduate, Avalon University School of Medicine; 6 Medical Student, American University of Integrative Sciences; 7 Pediatric, California Institute of Behavioral Neurosciences and Psychology, New York, USA; 8 Graduate, University College of Medicine and Dentistry, University of Lahore, Lahore, PAK; 9 Graduate, Pontifical Catholic University of Ecuador, Chagrin Falls, USA

**Keywords:** crohn’s disease, inflammatory bowel disease, steroids, relapse, mesalamine, colectomy

## Abstract

Crohn’s disease (CD) is a granulomatous inflammatory disease that can involve any part of the gastrointestinal tract, from mouth to anus. In most cases, it remits and relapses in the terminal ileum, requiring treatment via steroid boluses. In rare cases, however, CD can involve the pulmonary system presenting as dyspnea on exertion and dry cough. We present a case of a 38-year-old man who developed shortness of breath, cough, and wheezing for one month after a colectomy procedure due to recurrent toxic megacolon. He recovered and tolerated extubation successfully and was prescribed mesalamine as maintenance therapy for CD. His pulmonary symptoms after the colectomy, along with his imaging and pulmonary function tests, indicated pulmonary involvement in the lungs as a progression of the primary inflammatory bowel disease. After confirming this diagnosis, he was treated with oral high-dose steroids after successful diagnosis, and the patient’s symptoms improved dramatically. This case highlights often overlooked CD bronchopulmonary involvement in the postoperative period.

## Introduction

Crohn’s disease (CD) is a granulomatous inflammatory bowel disease (IBD) that can involve any part of the gastrointestinal tract. This disease usually relapses and remits. The main treatment goal for patients with CD treatment is to achieve and maintain remission. CD relapse is typically noted as the appearance of symptoms in patients taking chronic maintenance therapy. Up to the 25% of CD patients can have extraintestinal manifestations, such as erythema nodosum, arthritis, and pyoderma gangrenosum [1]. Pulmonary involvement is not a classic manifestation of CD, but it can occur, ranging from latent asymptomatic to severe clinical manifestations [2-4]. A variety of risk factors can cause progression of IBD to the lungs, and colectomy is a common cause [5]. We present a case of pulmonary involvement in CD presenting as dyspnea on exertion, cough, and wheezing after a surgical procedure.

## Case presentation

A 38-year-old African American man with a three-year history of CD presented to his primary care physician with concerns of dyspnea on exertion and dry cough for one month's duration following a partial colectomy and hospitalization due to toxic megacolon. During this previous hospital stay, he was extubated successfully and tolerated the procedure well. The patient was discharged on a stable maintenance dose of mesalamine. Postoperatively, he started experiencing progressive shortness of breath and a dry cough. The patient reported he had no history of asthma, chronic obstructive pulmonary disease (COPD), sarcoidosis, Goodpasture syndrome, or any chronic lung damage. On physical examination, wheezing was noted during lung auscultation. His laboratory values from his current visit are presented in Table 1.

**Table 1:** Clinical Laboratory Values on Presentation pH: potential of hydrogen; pCO_2_: partial pressure of carbon dioxide; HCO_3_: bicarbonate; O_2_: molecular oxygen

Analyte	Result
Hemoglobin	10.1 g/dL
Platelet count	150 x10^3^/µL
Peripheral blood smear	Elevated neutrophil count
C-reactive protein	12 mg/dL
pH	7.36
pCO_2_	45 mmHg
HCO_3_	23 mEq/L
O_2 _Saturation	90%
Lactate	6.6 mg/dL
Stool culture	Negative
Urine culture	Negative
Blood culture	Negative

Pulmonary function tests (PFTs) showed an obstructive pattern due to decreased forced expiratory volume in the first second (FEV1) as shown in Table 2.

**Table 2:** Pulmonary Function Test (Baseline Vs. After Corticosteroid Therapy) FEV1: forced expiratory volume in the first second; FVC: forced vital capacity

Function	Reference Value	Baseline	After Therapy
FEV1	3.78 L	3.6 L	3.7 L
FVC	4.8 L	5.3 L	4.6 L
FEV1/FVC	80%	67%	78%

**Figure 1:**
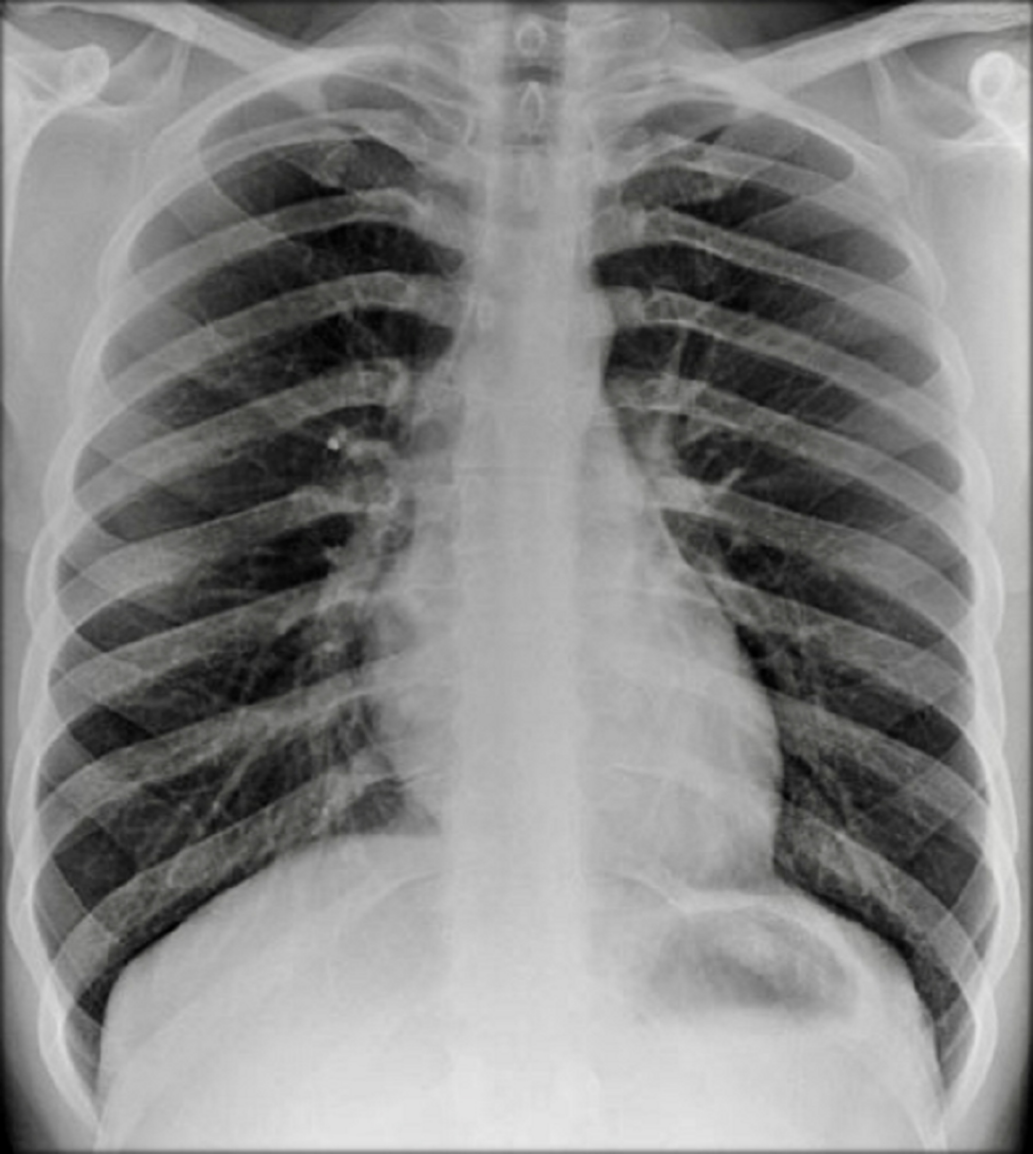
Chest x-ray

Bronchoalveolar lavage (BAL) showed high lymphocytic predominance (Table 3). A chest x-ray (Figure 1) and computed tomography (CT) scan were performed, the findings of which were unremarkable.

**Table 3:** Lymphocyte Typing in Blood and Bronchoalveolar Lavage

Lymphocyte typing	Blood	Bronchoalveolar Lavage
CD3+	76%	92%
CD4+	48%	52%
CD8+	22%	25%
CD4+/CD8+ (ratio)	2.16	2.08

Upon his first presentation, we prescribed empiric moxifloxacin due to a suspected postoperative respiratory infection. However, the patient remained clinically symptomatic after a week of antibiotic treatment. PFT results and his symptoms did not improve with the antibiotic trial. His history of CD for the past three years, a recent colectomy, his BAL findings, and his PFT results indicated his clinical symptoms were the result of CD involvement of his respiratory system. The patient was diagnosed with bronchitis due to CD, and a trial of high-dose oral steroids was prescribed, which yielded a dramatic improvement in his clinical symptoms. The patient underwent repeated PFTs, and his FEV1 returned to normal. We monitored him on an oral steroid and tapered the dose to the maintenance level gradually.

## Discussion

CD is a chronic granulomatous disease with a tendency to involve any part of the gastrointestinal tract. CD usually has a remission/relapsing pattern, and the most common location of CD involvement is the terminal ileum. However, CD can involve extraintestinal organs, such as the eyes, skin, joints, liver, and lungs [2]. One unusual location of CD is the pulmonary system. Respiratory involvement in IBD, including CD, was first described in 1976 by Kraft et al., who noticed that six patients developed bronchopulmonary involvement three to 13 years after the initial onset of IBD [6]. Other investigators have added context to the discussion to find risk factors causing pulmonary involvement in IBD. Camus et al. noted pulmonary involvement in CD is common in patients after a colectomy, presenting a few days to a few weeks following the surgical procedure [7]. Camus noted eight of 28 patients developed pulmonary symptoms after colectomy in IBD cases. In broad terms, CD can involve any part of the lungs—from the larger airways (e.g., the trachea) to the smallest airway (i.e., the alveoli).

The pathophysiology of respiratory involvement in CD is not clearly understood, but it may be linked to the shared embryologic descent from the primordial foregut for both the gastrointestinal and pulmonary systems [7]. Another possible mechanism for the emergence of respiratory tract involvement is that the gut and lungs both have mucosal-associated lymphatic tissue that holds a pivotal role in the mucosal response to pathogens [8]. This mucosal response to pathologic external antigens may be a nidus for the inflammatory changes seen in CD and result in the production of interleukins and tumor necrosis factor alpha, which in turn causes lung damage due to the development of reactive oxidative stress [5, 9].

Pulmonary involvement of CD can be latently asymptomatic or clinically symptomatic. Asymptomatic latent pulmonary disease in CD is very common and can be detected incidentally by abnormal screening tests, such as BAL and PFTs [10-11]. Although symptomatic pulmonary relapse of CD is rare, it can include upper airway obstructions, such as tracheal stenosis, trachea-bronchitis, chronic bronchitis, granulomatous bronchiolitis, and bronchiectasis [7, 12]. There are multiple risk factors for the relapse of CD in the lungs, but surgical procedures, such as colectomies, are a common risk factor for relapse [5]. Our patient’s history of CD and recent colectomy may have caused his condition to progress to pulmonary involvement. The differential diagnosis for lung disease in patients with a history of CD includes infections, COPD, chronic granulomatous disorders, and drug-induced lung injury [5]. Specific to this population, it is very imperative to differentiate between drug-induced lung damage and pure pulmonary relapse of CD. Drug-induced lung damage presents as a high eosinophil count on BAL instead of high lymphocyte counts normally seen in primary relapses of CD [13-14]. Our patient had high lymphocyte counts per our BAL findings depicted by an increased CD4/CD8 ratio as shown in Table 3.

A variety of pulmonary symptoms, a history of IBD, PFTs, imaging, bronchoscopy, and biopsy findings can aid in diagnosing pulmonary involvement of CD. Asymptomatic latent cases are usually detected by abnormal results in PFT and imaging findings. PFTs are recommended in asymptomatic latent cases as they can pick up lung involvement earlier than radiological investigations [15]. Physiological abnormalities in gas exchange can be seen in such latent cases and are often portrayed by low values of lung transfer factor for carbon monoxide [11]. Symptomatic pulmonary involvement presents with bronchitis, as it is one of the more common presentations [16]. PFT can show the same obstructive pattern in symptomatic cases. Like in our case, bronchitis usually presents unremarkable x-ray imaging results. Radiography findings may be negative in both asymptomatic and symptomatic cases [17]. Bronchoscopy is the diagnostic procedure of choice; it can show inflammatory changes, including redness, swelling, and patches in the tracheobronchial system. A biopsy can reveal non-caseating granuloma in the lungs [10]. Once patients are diagnosed as having CD with pulmonary involvement, steroids should be started as soon as possible. Aerosolized, oral, or intravenous steroids are extremely effective in treating the clinical symptoms of pulmonary involvement, and this effect can be strengthened by the normalization of PFT after treatment [5]. Our patient had clinically responded to steroid treatment, and this was supported by the normalization of his follow-up PFT as shown in Table 2.

## Conclusions

Our patient’s clinical presentation, laboratory, and imaging findings confirmed bronchopulmonary involvement in CD. Colectomy is a commonly linked risk factor for CD pulmonary involvement. Physicians should consider CD in their differential diagnosis if a patient has a history of CD and presents with pulmonary symptoms. Further research is necessary to investigate the pathophysiology of bronchopulmonary involvement in CD. Once the diagnosis is confirmed by a combination of clinical symptoms, imaging, and PFT results, high-dose steroids should be started and gradually tapered over the next few weeks. Patients often respond to the steroid treatment, and follow-up with repeated PFT is advised to detect the reversal of the obstructive pattern.
